# Theoretical and Experimental Research on Bubble Actuated Micro-Pumps

**DOI:** 10.3390/mi9050225

**Published:** 2018-05-09

**Authors:** Yang Qu, Junjie Zhou, Wei Wu

**Affiliations:** School of Mechanical Engineering, Beijing Institute of Technology, Beijing 100081, China; quyang@bit.edu.cn or quyang91@163.com (Y.Q.); wuweijing@bit.edu.cn (W.W.)

**Keywords:** micro pump, bubble actuated, theoretical model, experimental studies, bubble dynamics

## Abstract

Bubble actuated micro-pumps have great potential to be integrated into microfluidic systems to allow the independence of peripheral equipment. Previous studies on bubble actuated valveless micro-pumps have been mainly limited to experimental studies and numerical simulations due to the complex behavior of bubbles. In this paper, the construction of a mathematical model for a bubble actuated valveless micro-pump considering fluid dynamics, heat and mass transfer and bubble dynamics is described. A prototype was fabricated and tested to verify this theoretical model. The morphological evolution of the driving bubbles during the heating process was observed by a high-speed charge-coupled device (CCD) camera, the flow rate produced by the micro-pump under different working conditions was recorded and the test results were explained by the heat dissipation model. The model in this study was able to precisely predict the flow of micro-pumps in different drive modes. The principle behind defining the heating frequency and the duty cycle based on the pump chamber volume was determined. The study shows the mechanism of bubble controlling and the good prospects of bubble actuated valveless micro-pumps.

## 1. Introduction

Micro-pumps are widely used in situations in which small and precise volumes of fluids are driven and controlled for chemical, biological or medical engineering systems. Though the micro-fluidic chips can be very small and easy to carry, the existing micro-pumps generally require external devices, which are costly, limiting the application of the chips. Previously, different reviews have been conducted that have summarized several works in this area [1,2,3]. Micro-pumps can be driven by mechanical components, such as pistons, diaphragms or gears. A pair of nozzle/diffuser elements generally acts as an incomplete check valve in these micro-pumps [4]. Some non-mechanical principles can also be used to actuate the pump, such as electro-hydrodynamic [5], light [6], bubble [7,8,9], or magnetic [10,11,12]. Among them, bubble actuated micro-pumps have advantages such as low cost and production simplicity, even on a large scale, since they have no moving parts. In addition, bubble actuated micro-pumps can be driven with relatively low voltage, while off-the-shelf piezoelectric micro-pumps require more than 100 volts, which is an important limitation. This study shows that the driving voltage of bubble actuated micro-pumps can be as low as 6 volts, which means that it can be actuated by dry batteries, eliminating the need for transformer circuits.

Compared to the commonly-used peristaltic micro-pumps [13,14] and shape-memory alloy (SMA) actuated micro-pumps [15], thermal bubble actuated micro-pump does not need a high voltage or temperature to drive the fluid, which makes it cheaper and more compact. However, its flow rate and controllability are not as good as the other two types of micro-pumps. Although the surface tension driven pump allows low voltage operation as well as low power consumption [16], similar to the current work, it needs a more complex structure for its continuous electrowetting mechanism.

The idea of pumping liquid with a bubble can be traced back to 1995 when Ozaki [17] asymmetrically heated a single bubble in a pipeline to make the bubble expand in one direction, driving fluid flow directionally. Reference [7] used multi-bubble methods, in which one bubble acts as a valve and the other one expands to pump the fluid; with the plurality of air bubbles, coordinated work is needed and the bubbles are difficult to control. Reference [18] used a platinum micro-heater to heat the water in a micro-channel and found that the heating signal pulse width and mass flow had little influence on the boiling inception time. A thermodynamic model of bubble growth and collapse in micro-channels is described in reference [19]. A more practical bubble actuated micro-pump was invented by Tsai et al. [20], who developed a bubble-driven valveless micro-pump consisting of a diffuser, a nozzle, a pumping chamber and a heater. However, this was only the experimental work. Reference [21] fabricated and tested a similar micro-pump under several conditions. However, it is interesting that their results were quite different from Tsai’s results, due mainly to the much bigger chamber volume of their pump. They gave a possible explanation for this difference, suggesting that the flow is influenced by the volume of the pump chamber and the duty cycle and frequency, but they did not make a quantitative calculation to show the specific relationship between the flow and the influencing factors. Deng [22] established a numerical model for bubble-driven valveless micro-pumps.

Due to limitations from the bubble dynamics, impact of the gas–liquid two-phase heat transfer and coupling effect of pump chamber and inlet/outlet resistance characteristics, previous studies of thermal vapor bubble driven valve-less micro-pump mechanism have mainly been numerical simulations and experimental studies. These are time-consuming and laborious. Therefore, to carry out more in-depth studies with a lower computational cost, a theoretical model based on the lumped parameter approach of a bubble driven valveless micro-pump is proposed in this paper. This model uniquely considers the bubble dynamic model, the fluid dynamic model and the gas thermodynamic model. The proposed model, validated by the experimental study, allows more theoretical understanding of micro-pump mechanisms and offers the basis for the design of more efficient bubble pumps.

## 2. Theoretical Modeling

**Figure 1:**
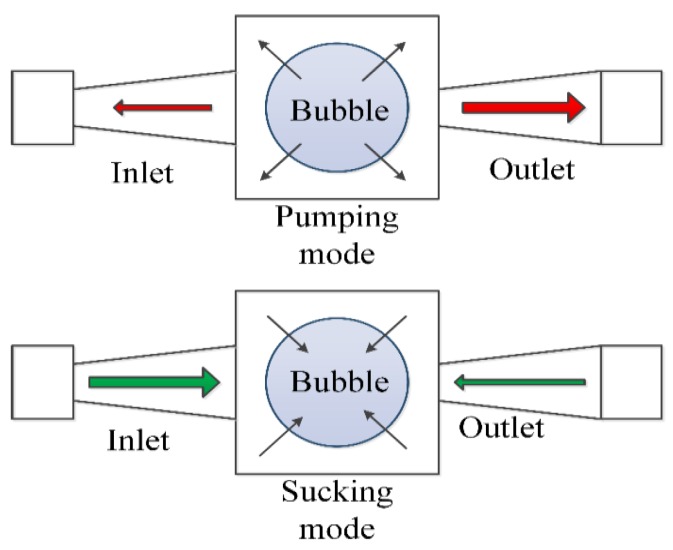
Working principle of the bubble actuated valveless micro-pump.

As can be seen in Figure 1, there are two working modes for this pump: pumping mode and sucking mode. Due to the different resistance of inlet/outlet ports, there is greater flow in the outlet port than the inlet port for the pumping mode, and on the contrary, there is more flow in the inlet port than the outlet port for the sucking mode.

The model of the micro-pump mainly consists of two parts: the fluid dynamic model for the pump chamber and the bubble dynamic model for the gas bubbles. The fluid dynamic model is the same as other types of valveless micro pumps. The major factors we consider are the pressure in the pump chamber and the flow resistances of the nozzle and the diffuser.

The diffuser and the nozzle operate as incomplete one-way valves—when the vapor bubble expands, the liquid flows out of the chamber, and the inlet acts as a nozzle with a relatively bigger resistance, while the outlet acts as a diffuser with a smaller resistance. Therefore, the flow of the outlet is larger than that of the inlet. However, when the vapor bubble contracts, the liquid flows into the pump chamber, and this time, the inlet acts as a nozzle and the outlet acts as a diffuser. The flow of the outlet is smaller than that of the inlet, thus leading to a net flow from the inlet towards the outlet.

The flow rate (*Q*) depends on the coefficients of pressure loss (ξ) of the nozzle or diffuser and the pressure difference on the nozzle or diffuser.

In pumping mode, the flow of the inlet and the outlet can be calculated by the equations below:

(1) $$Q_{1}=-A\sqrt{\frac{2\left(p_{c}-p\right)}{\rho_{L}\xi_{n}}}$$

(2) $$Q_{2}=A\sqrt{\frac{2\left(p_{c}-p\right)}{\rho_{L}\xi_{d}}}$$

where, *Q*_1_ is the flow rate of the inlet. *Q*_2_ is the flow rate of the outlet. *p* is the pressure outside the pump chamber. *p*_c_ is the pressure in the chamber. $\rho_{L}$ is the density of the liquid. $\xi_{n}$ is the pressure loss coefficient of the nozzle. $\xi_{d}$ is the pressure loss coefficient of the diffuser. *A* is the area of the narrowest section of the nozzle or diffuser.

In sucking mode, the flow of the inlet and the outlet can be calculated by the equations below:

(3) $$Q_{1}=A\sqrt{\frac{2\left(p-p_{c}\right)}{\rho_{L}\xi_{d}}}$$

(4) $$Q_{2}=-A\sqrt{\frac{2\left(p-p_{c}\right)}{\rho_{L}\xi_{n}}}$$

**Figure 2:**
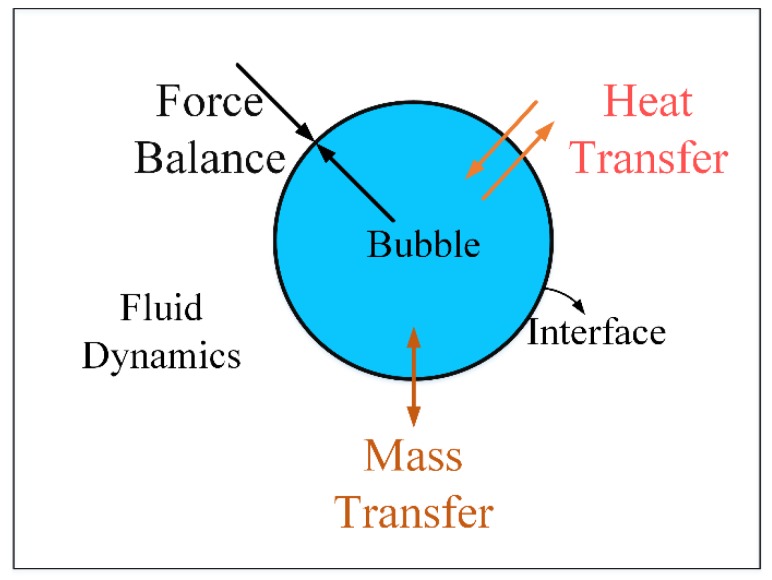
Model of the bubble.

The model of the bubble takes bubble dynamics and heat and mass transfer into consideration, as shown in Figure 2.

Bubble dynamics equation: according to the Rayleigh–Plesset equation, the change of *R* is in accordance with Equation (5):

(5) $$\rho \left(R\ddot{R}+\frac{3}{2}{\dot{\;R}}^{2}\right){\;=\;p}_{b}-p_{c}-\frac{2\sigma }{R}-\;4\mu \;\frac{\dot{R}}{R}$$

where, *R* is the radius of the bubble, *p_b_* is the pressure in the bubble, σ is the surface tension, and *μ* is viscosity of the liquid.

(6) $$\frac{{dp}_{c}}{dt}=\;\frac{E_{L}}{V_{c}-V_{b}}\left(Q_{2}-Q_{1}+\frac{dV_{b}}{dt}-\frac{1}{\rho_{L}}\frac{dm}{dt}\right)$$

where, *V*_c_ is the volume of the pump chamber, *V*_b_ is the volume of the bubble, d*m* is mass of the liquid which transfers into gas. $E_{L}$ is the volume modulus of the liquid.

Mass transfer equation: According to the first law of thermodynamics, the energy input is used in two ways—for the evaporation of the liquid and the expansion of the gas:

(7) $$dmh_{lv}=P-p_{b}dV_{b}-hS\Delta T$$

*h_lv_* is the vaporization latent heat of the liquid. *P* is the heating power, *h* is the convective heat dissipation coefficient for which a value of 700 W/m^2^·K, based on experience, was used for this study. *ΔT* is the temperature difference across the bubble surface. Assuming the bubble exists at the boiling point of ethanol while the pump is working at room temperature, then the temperature difference is assumed to be 40 °C.

Only the surface area (*S*) of the bubble is variable in Equation (8), and this is dependent upon the bubble radius *R*.

When the bubble volume is too small, the error will be too large for the numerical model to calculate, A minimum bubble radius of 1 × 10^−20^ m is defined. When the bubble radius is less than the minimum radius and the input power is 0, the volume of the bubble stays constant.

For vapor, according to the ideal gas state equation, there is

(8) pV =mRgT

Derivate the total differential of both sides; note that phase changing is an isothermal process, so d*T* = 0, and there is

(9) $$dp_{b}=\frac{1}{V_{b}\left(RgTdm\;-\;p_{b}dV_{b}\right)}$$

Thus, given the heating power and the temperature of the environment, the pressure of the bubble can be calculated with the bubble dynamics model. Then, the flow of the pump can be determined with the fluid dynamics model.

## 3. Experimental Study

To verify the validity of the model, a prototype was manufactured, and the flow produced by it was measured with the testing system described below.

### 3.1. Description of Testing System

**Figure 3:**
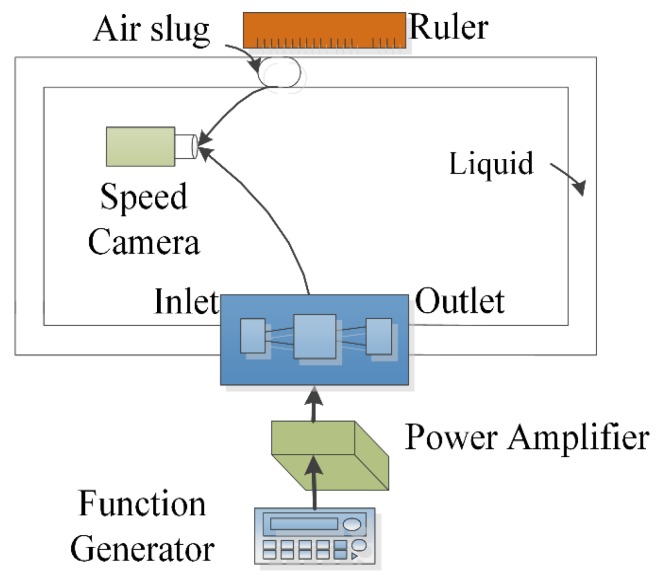
Schematic of experimental apparatus.

The experimental setup included a signal generator, a power amplifier to heat the bubble by the connected resistance wire above the pump chamber, the test bubble pump, the flexible tubes, the speed camera to record the bubble operation and a ruler to measure the movement of the air slug, as shown in Figure 3. To actuate the pump, a fluctuating current was generated to heat the chamber to generate the bubble. When the pump started to work, the air slug was expected to move from outlet towards the inlet.

Alcohol was chosen as the working fluid due to its appropriate boiling point, extensive application, good availability and safety.

A square wave signal with a frequency range of 10 Hz to 200 Hz was generated by a signal generator and then amplified by a DC amplifier (FPA0510S, Feeltech Co. Ltd., Zhengzhou, China). The voltage of the input signal was set as 14 V, but according to the measurement with the real-time data acquisition card, we found that the maximum instantaneous voltage actually applied on the micro-pump was only 6 V. This is mainly due to the larger internal resistance of the signal source.

A Photron Fastcam SA3 Speed Camera (Photron, Tokyo, Japan) was used to obtain the image of the bubble’s morphological changes.

**Figure 4:**
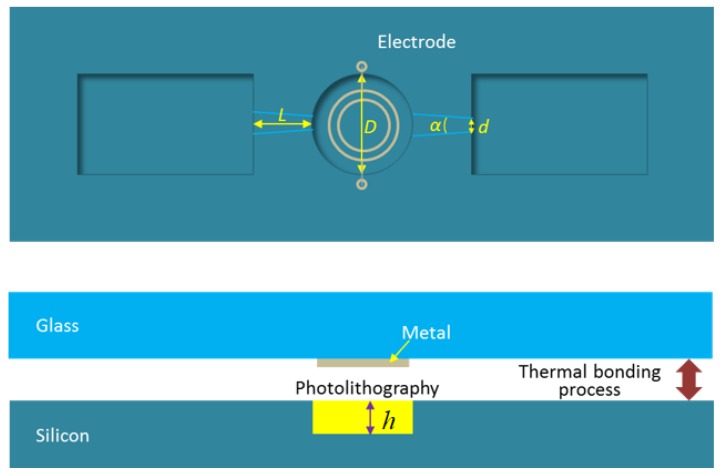
Fabrication process of the micro heater. (*D* and *h* are the diameter and the depth, respectively, of the pump chamber; *L* indicates the length of inlet/outlet ports; *d* is the diameter of the small end and *α* is the convergence angle of the inlet/outlet ports).

The fabrication process of the micro-pump is shown in Figure 4. The pump chamber was generated by a deep reactive ion etching (DRIE) process on a silicon wafer. The etching depth (*h*) was 200 nm. The heater was manufactured by a metal film sputtering process on a glass wafer. The silicon wafer was bonded together with the glass wafer. The heater was manufactured by an aluminum film sputtering process, and there were two very thin wires extracted by the imbedded electrode in the silicon layer. The resistance of the heater was 30 Ω, and with a voltage of 6 V, the instantaneous power of the heater was 1.2 W. The average power of the pump depended on the duty cycle in each cycle. For example, if the duty cycle was 40%, the average power of the micro-pump was 0.48 W.

**Figure 5:**
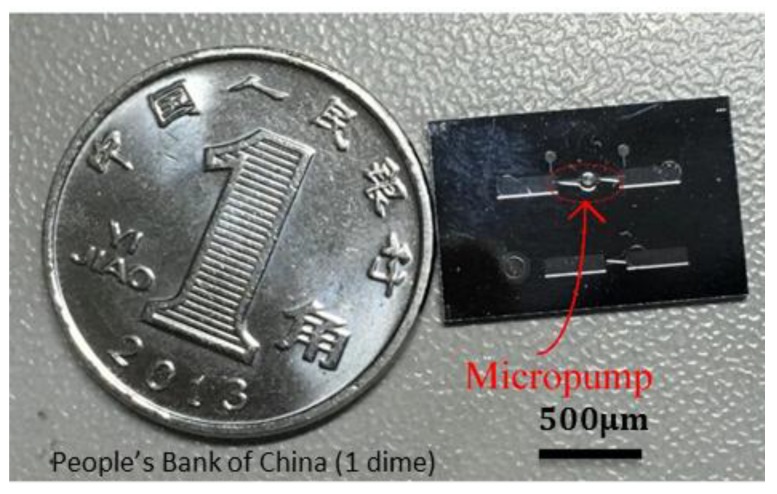
Micro-pump compared with a coin.

The diameter of the pump chamber (*D*) was 1 mm, the narrowest width of the nozzle and the diffuser (*d*) was 30 μm, the diverging angle of the nozzle and the diffuser was 14°, and the length (*L*) is 1 mm. The size of the micro-pump is compared with a coin in Figure 5.

**Figure 6:**
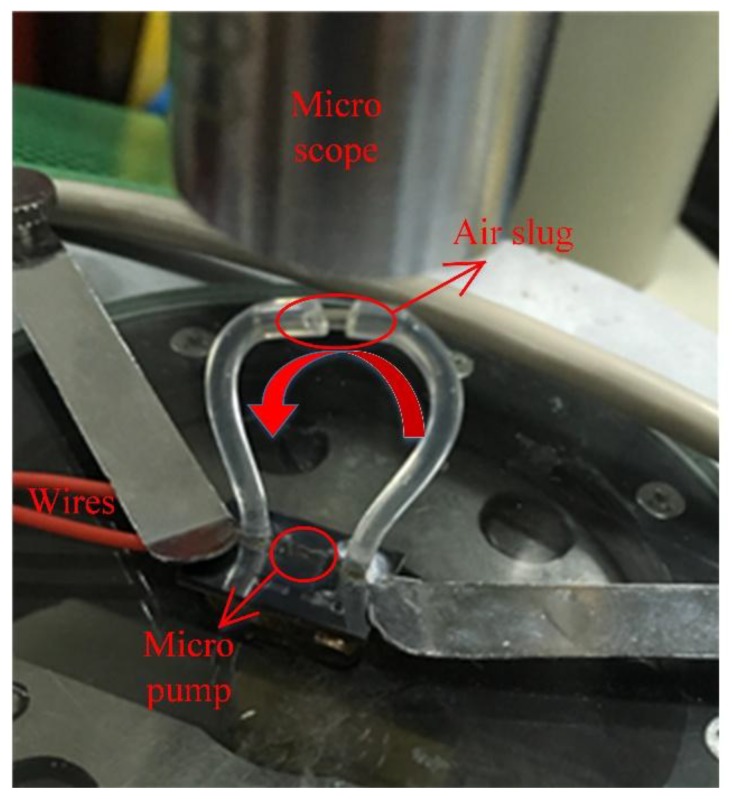
Measurement of the flow rate.

Because the pump and channels were too small, an indirect method was adopted to measure the flow rate. As can be seen in Figure 6, there were two flexible tubes connected to the inlet and outlet ports of the pump, and a thin tube between them with an air slug. The air slug was used to mark the flow in the channels. The diameter of the section of the transparent conduit was 0.8 mm. By recording the time during which the air slug moved from one end of the section conduit to the other end, the flow rate was calculated.

### 3.2. Work Process Description

**Figure 7:**
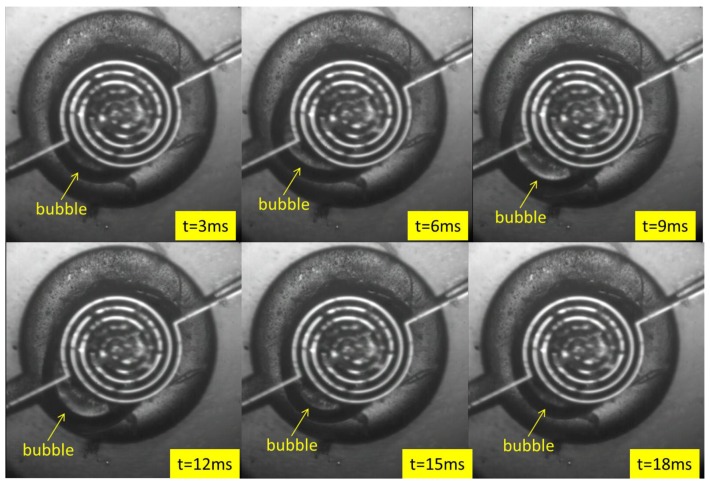
Operation sequence of the bubble actuated micro-pump at a heating frequency of 25 Hz and at a 20% duty cycle (the black arc is the outline of the bubble).

Figure 7 clearly shows that the volume of the bubble gradually increased and then gradually decreased during one operating cycle. Due to the obstruction of the heater, it cannot be observed when the bubble volume is too small. The response of the bubbles was very fast, the speeds of bubble expansion and contraction were different, and the growth and reduction of the bubbles were asymmetric, which could be caused by the liquid flow. For more details, one can also see Video S1.

## 4. Results

**Figure 8:**
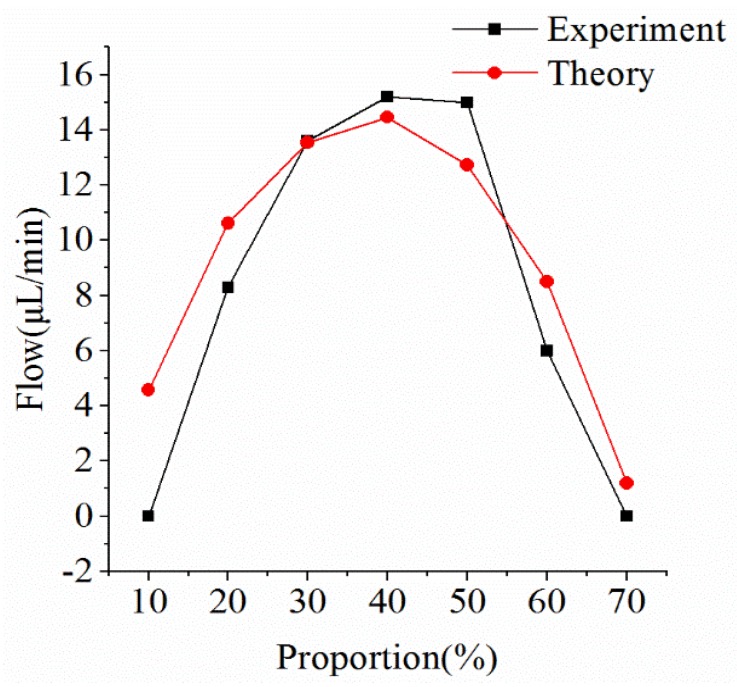
Comparison of theoretical flow and experimental flow at a heating frequency of 50 Hz.

Figure 8 shows that the flow varied along with the duty cycle when the heating frequency was 50 Hz. Under this condition, the actual flow of the micro-pump was very close to the simulation result. The maximum flow was 15 μL/min, which occurred when the duty cycle was 40%, validating the mathematical model. In the simulation model and in the experiment, the flow increased as the duty cycle increased, until it reached a maximum, and then the flow decreased.

The reason for this trend will be discussed below.

**Figure 9:**
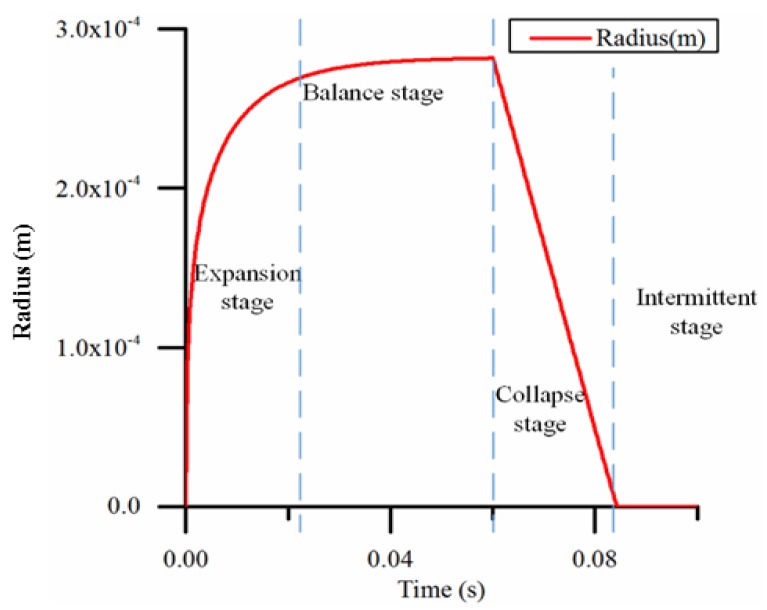
A typical result of the bubble radius during one cycle with adequate time.

Figure 9 shows the simulation results of the change in bubble radius in a heating cycle. When the heating and cooling times are adequate, the process of the bubble volume change can be divided into four typical stages:

1. Expansion stage: At the beginning of heating, the bubble volume expands rapidly. At this time, the surface area is small; therefore, the heat dissipation effect is obscure.
2. Balance stage: when the bubble volume reaches a certain amount, the surface area increases, the heat dissipation rate gradually increases, and the heating power and cooling power are in balance so that the bubble volume remains constant.
3. Collapse stage: When heating stops, the bubble volume gradually decreases as the heat dissipates.
4. Intermittent stage: At this stage, the bubble has disappeared and the next heating stage has yet to begin.

Due to differences of the heating frequency and proportion (percentage of the heating time in one pump cycle) in the actual work process, one or two of the four states of the process may not appear.

**Figure 10:**
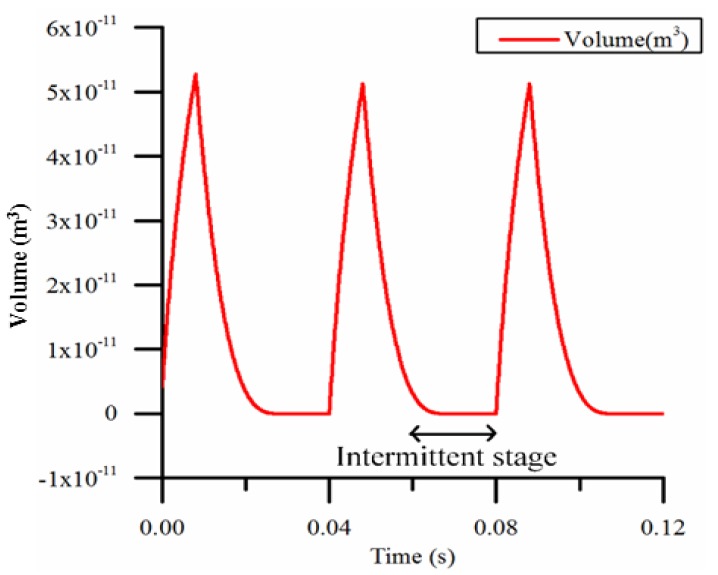
The volume of bubble calculated by the model when the duty cycle is 20% and the heating frequency is 25 Hz.

As shown in Figure 10, when the duty cycle is too small (for example, 20%) and the frequency is too low (for example, 25 Hz), the intermittent period during which the micro-pump is not working is too long. The pump is making no contribution to the flow, so the flow decreases as the effective working time decreases.

**Figure 11:**
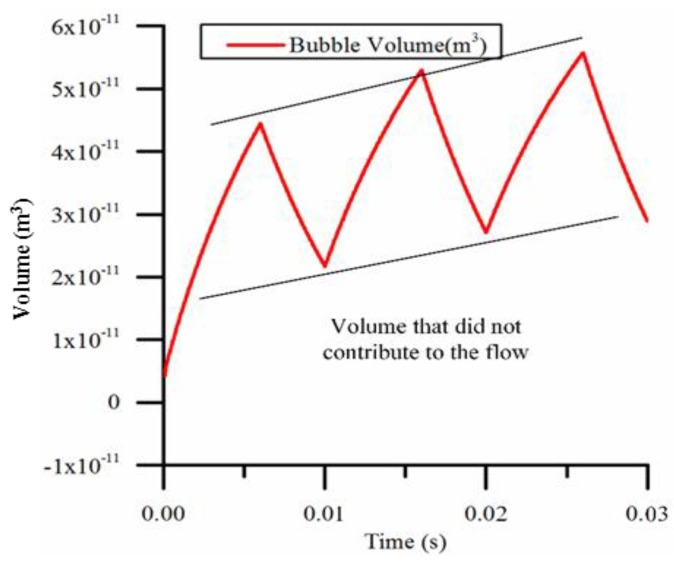
The bubble volume calculated by the model when the duty cycle is 60% and the heating frequency is 100 Hz.

As shown in Figure 11, when the duty cycle is too large (for example, 60%) and the frequency is too high (for example, 100 Hz), the time is too short for the bubble to cool down completely and collapse. In this case, the volume of the pump chamber is not utilized effectively. The volume change decreases and the effective working volume decreases, resulting in decreased flow, compared with the case in which the bubble contracts sufficiently.

In addition, when the heating power is relatively high, the volume of the pump chamber determines the maximum volume of the bubble. In the case of low heating power, heating power and heat dissipation determine the size of the bubble.

Based on the rules above, we can see that for a defined pump chamber volume and heating frequency there will be a suitable duty cycle to achieve maximum flow. With high frequency, a lower duty cycle is needed to achieve a greater working flow due to the short intermittent heating period and subsequent short heat dissipation time.

**Figure 12:**
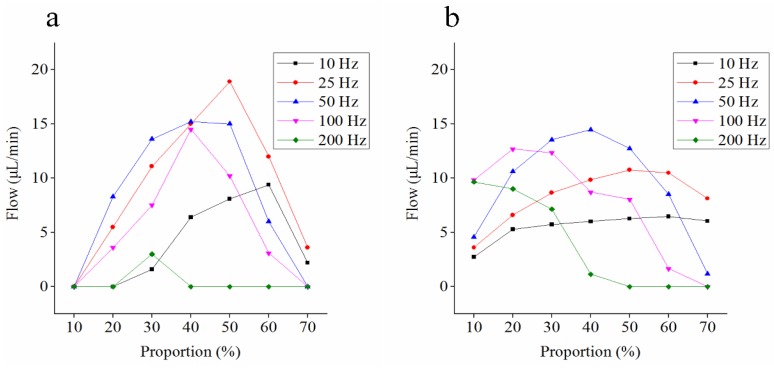
Comparison of the theoretic flow (**a**) and experimental flow (**b**) under different conditions.

We also calculated and verified the working results of micro-pump at different frequencies, as shown in Figure 12. The results show that the model can predict the working effect of micro-pump at most frequencies, especially with high duty cycles, such as 50–70%.

However, when the duty cycle is small, the model does not appear to be very accurate. The inaccuracy is mainly the result of not considering the nucleation process in this model. Former studies have shown that the liquid should be heated over the boiling point to generate a bubble. When the temperature of the liquid has already reached the vaporization temperature, the surface tension has a very large pressure on the gas when the bubble radius is very small, and tiny bubbles soon disappear. A very short heating time is insufficient to produce bubbles, and it is even insufficient to keep the temperature in the pump chamber at the boiling point. However, in this model, it is assumed that the temperature is kept at the boiling point.

There are observed phenomena that may have contributed to the error.

If the micro-pump starts to work in a cold environment, or the cooling conditions deteriorate, the ambient temperature will rise, resulting in the maximum volume of bubbles in each cycle gradually becoming larger and larger, and the bubble volume change will be similar to Figure 11. The model does not take into account changes in ambient temperature, and we assume that the temperature difference between bubbles and the outside world is a constant value, which is obviously not realistic. In fact, during the stage in which the micro-pump has just begun to work, the ambient temperature must rise. However, the final ambient temperature will eventually become stable at a specific value. This model can be used when the ambient temperature is stable.

## 5. Conclusions

A lumped parameter model was constructed for the thermal bubble actuated valveless micro-pump, and a prototype was fabricated. Experimentation demonstrated that this model is able to predict the flow rate of the micro-pump under different working conditions. The changes of the hot bubbles throughout entire heating cycle were observed, and the changes of the bubbles under different heating conditions and the influence on the flow rate were analyzed. It was determined that the heating frequency and the duty cycle should be defined based on the size of the pump chamber.

This study shows that the thermal bubble-driven valveless micro-pump is a controllable device that requires very low drive voltage and power and has good development prospects. It is possible to apply this model in microfluidic chips to replace the external pumps and to improve the system integration.

In the future, a more precise model considering the nucleation process and the change in ambient temperature will be developed and the applicability of that model with a variety of pump chamber volumes will be tested. In addition, the method to stabilize the flow rate of pump will be studied.

## Supplementary Materials

The following are available online at http://www.mdpi.com/2072-666X/9/5/225/s1, Supplemental Video S1: Bubble operation.
